# Optic Neuritis in a Child With Poorly Controlled Type 1 Diabetes Mellitus: A Case Report

**DOI:** 10.7759/cureus.33474

**Published:** 2023-01-07

**Authors:** Anood Alassaf, Khalid Mohamed, Abdullah Al Otaiby, Mohammad Al Wraidat, Abdulqadir J Nashwan

**Affiliations:** 1 Pediatrics Department, Hamad Medical Corporation, Doha, QAT; 2 Pediatrics Department, Sidra Medicine, Doha, QAT; 3 Neurology Department, Sidra Medicine, Doha, QAT; 4 Internal Medicine Department, Hamad Medical Corporation, Doha, QAT; 5 Nursing Department, Hamad Medical Corporation, Doha, QAT

**Keywords:** pediatric diabetes, diabetic eye disease, ophthalmology, diabetes type 1, optic neuritis

## Abstract

Type 1 diabetes stands among the most prevalent endocrinological diseases in the pediatric age group. The incidence rate continues to rise globally. Optic neuritis has been described in the literature in association with type 2 diabetes; however, cases of optic neuritis with type 1 diabetes are very few. Here we describe a rare case of a 15-year-old patient with type 1 diabetes mellitus presenting with optic neuritis. Due to the hyperglycemia that steroids can induce in some patients, management with steroids can be difficult. A multidisciplinary team approach is required to ensure that these patients' optic neuritis is properly handled while avoiding steroid side effects.

## Introduction

Type 1 diabetes stands among the most prevalent endocrinological diseases in the pediatric age group. The incidence rate continues to rise globally, reaching up to 2.9 new cases per year per 100 000 persons below 15 years of age [1]. Looking at the gulf region, the prevalence of diabetes appears to be one of the highest globally. The percentages of diabetic patients (both type 1 and type 2) in five countries located in the gulf peninsula (Kuwait (21.1%), Qatar (20.2%), Saudi Arabia (20.0%), Bahrain (19.9%) and UAE (19.2%)) rank among the highest 10 countries in the world. In 2020, Qatar particularly reported an incidence rate of Type 1 diabetes mellitus (T1DM) of 38.05 per 100,000 individuals [2]. The increased number of T1DM cases has been reflected in the number of diabetes-related complications and associated diseases that come to medical attention, mainly ocular diseases. Some of these diseases are well known, like diabetic retinopathy, and others are rare; for example, Anterior ischemic optic neuropathy (AION) and diabetic papillopathy. Optic neuritis has been described in the literature in association with type 2 diabetes [3]; however, cases of optic neuritis with type 1 diabetes are very few. Here, we describe a rare case of a 15-year-old patient with type 1 diabetes mellitus presenting with optic neuritis.

## Case presentation

The patient is a 15-year-old girl who was diagnosed with type one diabetes mellitus in 2013. She has positive glutamic acid decarboxylase (GAD) antibodies and highly positive islet insulin antibodies. Her screening at the time of diagnosis was negative for celiac and autoimmune thyroid diseases. Her height was 138 cm, which is on the 0.02 centile, and her weight was 44 kg (on the 14^th^ centile) with no recent loss. Menarche occurred two months back at Tanner's stage 4 of breast development. She was on a basal-bolus insulin regimen, receiving 20 glargine units in the evening and three fixed doses of Aspart insulin (five units before breakfast, 10 units before lunch, and five units before dinner). This regimen was the most suitable for her as she had great difficulty doing carbohydrate counting and giving insulin accordingly. Despite that, she had poor compliance due to social reasons, leading to unsatisfactory glycemic control. Her latest HbA1C at that time was 13.9% (Table 1). After multiple missed clinic visits, the patient attended her first annual diabetic retinopathy screening after nine years of diabetes onset, in which her optic disc and fundus color photos showed early hard exudate in the background of the fundi in both eyes, with normal optic discs and no evidence of retinal microvascular abnormalities at this stage. Visual acuity was 6/29 in both eyes using Snellen’s chart. She was scheduled for another annual ophthalmological screening test but missed her appointment. Her most recent annual diabetic nephropathy screening was significant for microalbuminuria (Albumin: Creatinine ratio 9.6 mg/mmol). Since her diagnosis, she has been admitted to the hospital four times with moderate diabetic ketoacidosis.

**Table 1:** Laboratory results

Test	Result	Normal range
HbA1C	13.9%	Normal < 5.7% Pre-diabetes 5.7% - 6.4%
ESR (erythrocyte sedimentation rate)	53 mm\hr	< 13 mm\hr
Albumin: Creatinine ratio	9.6 mg\mmol	< 3 mg\mmol

She presented to the emergency department with left vision loss for three days. She initially had blurry vision and difficulty distinguishing colors, which progressed to partial vision impairment, described by the patient as not being able to see well or identify the detailed parts of the objects close to her. No other symptoms were noted. Ocular pain was absent; however, she admitted she had some degree of discomfort with eye movement. She has no history of preceding viral illness or head trauma and no weakness or sensory changes in her limbs.

Detailed clinical examination of the pupils showed a relative afferent pupillary defect (RAPD) in the left eye. Red reflex was present bilaterally. In addition, she had impaired color vision and brightness perception in the left eye. Her visual acuity in the left eye was 6/30 using Snellen’s chart (0.7 LogMar), while the visual acuity in the right eye was 6/15 with Snellen’s Chart (0.44 LogMar). Visual field examination by confrontation showed left inferior nasal quadrantanopia. Retinal examination showed severe and diffuse optic disc swelling (grade 4 optic disc edema using the Frisen's scale), mild hard exudate in the background of both fundi, and microaneurysms in the left eye, confirming the diagnosis of left optic neuritis with features of diabetic retinopathy. The right optic disc looked normal.

**Figure 1:**
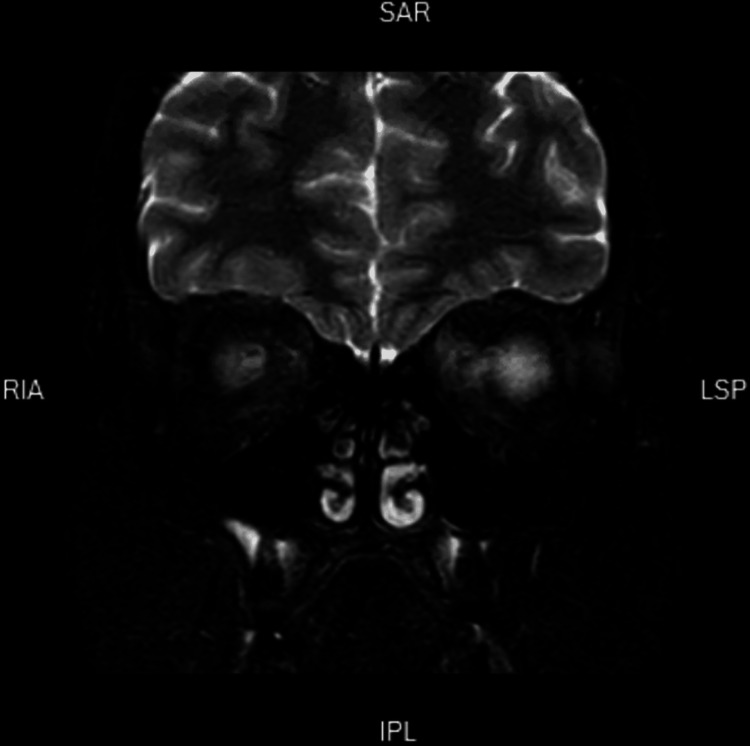
T2 brain MRI (coronal section) demonstrates enhancement of the pre-chiasmatic segment of the left optic nerve.

The patient was admitted to the neurology ward, where a brain MRI was done. The MRI showed minor focal asymmetrical enhancement of the left pre-chiasmatic optic nerve segment with no associated supratentorial parenchymal or posterior fossa demyelinating features (Figure 1). Blood tests showed an elevated ESR of 53 mm/hr. Complete blood count test, electrolytes, kidney, and renal function were normal. Testing for myelin oligodendrocyte glycoprotein (MOG) and AQP-4 (aquaporin-4) antibodies came back negative for both.

The patient received Methylprednisolone 1g intravenously, daily (divided into four doses) for three days in the hospital and was discharged home with oral prednisolone 1 mg/kg/day for 14 days. Her retinal examination in the ophthalmology clinic after two weeks after discharge showed a normal appearance of the optic disc.

**Figure 2:**
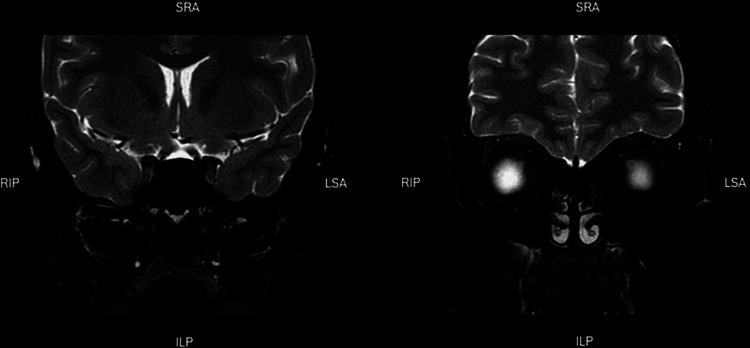
Coronal T2W MRI of the brain that was repeated after three weeks after hospital discharge. The optic nerves appeared of normal caliber. There was a stable subtle asymmetrical enhancement of the prechiasmatic segment of the left optic nerve. No other significant enhancement of the optic pathway noted.

Direct and consensual pupillary reflexes were normal, with no evidence of rapid afferent pupillary defects on both sides. However, the latest findings of mild retinal hard exudate with microaneurysms were persistent. Head MRI was repeated after three weeks from discharge. The optic nerves appeared of normal caliber. There was a stable subtle asymmetrical enhancement of the prechiasmatic segment of the left optic nerve. No other significant enhancement of the optic pathway was noted .no new intracranial lesions (Figure 2). No focal intracranial lesions or areas of altered signal intensity were seen.

While in the hospital, the patient had elevated pre-prandial blood sugar readings ranging between 14 and 18 mmol/L. The endocrinology team increased her basal insulin to 27 units and started her on Aspart insulin as per the carbohydrate counting regimen, with Intracameral Ranibizumab (ICR) 1:6 gm, Insulin Sensitivity Factor (ISF) 1:3mmol, and a blood sugar target of 7 mmol/L. The patient and the caregiver received dietary counseling and support from social services. On discharge, her glargine dose was adjusted to 25 IU, and she showed readiness to do insulin correction based on meal carbohydrate counting.

The patient was seen in the endocrinology clinic after two weeks after discharge. She stated that she was compliant with basal insulin and carbohydrate counting for meals most of the time. She was using a personal continuous glucose monitoring device which showed that the time in range (TIR) was only 17%, while the time above range (TAR) was 72%. As the patient has just finished her 14 days course of oral steroids, the endocrinologist taking the caregiver’s wishes into consideration, decided to assess the patient again after two weeks before making further adjustments. The latest endocrinology appointment one month after discharge showed that most of her glucose readings were within the normal range; therefore, she was kept on 25 units of basal insulin, and no changes were made to her ICR or ISF.

## Discussion

Type 1 diabetes mellitus is the commonest metabolic disorder in children and adolescents [1]. It is widely known for its numerous complications, which mainly occur in the case of poor compliance with treatment. One of the most feared complications is ocular involvement. While the most common ocular complication is diabetic retinopathy, other less common ocular diseases can also be present, including cataracts, anterior ischemic optic neuropathy, diabetic papillopathy, and extraocular muscle palsy. In addition to being associated with many ocular diseases, diabetes is a known risk factor for others; for example, primary glaucoma, neovascular glaucoma, and ocular ischemic syndrome [4].

Optic neuropathy is a term used to describe a group of ocular diseases related to the optic nerve. It includes ischemic neuropathy, optic neuritis, and optic atrophy. Amongst the main types of optic neuritis, retrobulbar neuritis and diabetic papillopathy are more often associated with demyelinating diseases, while peri neuritis and neuro retinitis are more commonly associated with infectious and inflammatory diseases. Optic neuropathy has been described in patients with type one diabetes; however, the incidence is only 1.54%. Compared to ischemic neuropathy, optic neuritis is less common, with an incidence of 3.7% of the total optic neuropathies [3].

The presentation of optic neuritis can be variable in severity, with some differences between pediatric and adult patients. Our patient mainly presented with acute unilateral vision loss, with a mild degree of discomfort sensation in the eye. In patients with optic neuritis, pain is a common feature of vision loss, and it can range from mild discomfort to severely intense pain. In the majority of patients, the pain is mild to moderate, subacute, retro-orbital, and frequently worsened with eye movement. The optic neuritis treatment trial (ONTT) has found that 95% of patients presented with unilateral vision loss while 92% had associated retroorbital pain that frequently worsened with eye movement12. While most cases are unilateral, optic neuritis involving both eyes, either simultaneously or sequentially, is more frequently seen in pediatric patients, especially those with myelin oligodendrocyte glycoprotein IgG (MOG-IgG) or anti-aquaporin-4 (AQP4) IgG antibodies in their blood [5].

The patient reported in our case had poorly controlled type 1 diabetes with diabetic retinopathy, which we believe has led to optic neuritis. Though the exact mechanism is not very well understood, Appen et. al. proposed that patients with diabetes may sustain a local optic disc vasculopathy due to chronically high levels of blood sugar, resulting in the development of diabetic papillitis [6]. In 1926, Francis and Koeing published a case report about retrobulbar neuritis in diabetes. They suggested that the ketone bodies are directly responsible in such cases [7].

Evaluation of diabetic patients with optic neuritis, especially in the pediatric age group, should focus on ruling out possible underlying causes of optic neuropathy before associating it solely with diabetes. In clinically stable patients, it begins with a thorough review of systems and neurologic examination looking for signs of multifocal or systemic diseases. The following blood tests should be performed: complete blood count (CBC), anti-neutrophil cytoplasmic antibody (ANCA), and anti-nuclear antibody (ANA). Anti-AQP4-IgG levels can be ordered if neuromyelitis optica, which is uncommon in pediatric patients, is suspected. It tests the antibodies against aquaporin-4, which is a serum marker with high sensitivity and specificity of neuromyelitis optical [8], a diagnosis that can be considered in the proper clinical context. To confirm optic nerve enhancement, brain and orbital MRI, with and without contrast, should be done. The MRI will also identify space-occupying lesions, meningeal enhancement, and inflammatory or demyelinating lesions elsewhere in the brain if present. Spine MRI might not be indicated without spinal cord signs unless AQP4-IgG testing is positive [9].

Regarding the treatment of pediatric optic neuritis, most neurologists recommend giving three to five days of intravenous methylprednisolone (4-30 mg/kg per day) while the patient is in the hospital, followed by prolonged oral corticosteroid tapering over a period of two to four weeks to avoid recurrence, which occurs more frequently in children compared to adults [10].

However, treating the acute phase of optic neuritis with corticosteroids has been a controversial subject. ONTT is a famous study that examined this idea. It found that steroids can hasten visual recovery and may decrease the possibility of being diagnosed with multiple sclerosis after two years. Interestingly, the outcome of the final visual recovery was unchanged. Our patient was commenced on corticosteroids aiming to expedite vision improvement and alleviate her anxiety, taking into consideration the challenges caused by steroid side effects on her glycemic control [11]. She received a high dose of IV methylprednisolone for three days in the hospital and 14 days of oral prednisolone given at home. Fortunately, she recovered with a favorable outcome.

It must be kept in mind that this treatment is not free of side effects, particularly steroid-induced hyperglycemia, which can be troubling in diabetic patients. Glucocorticoids increase insulin resistance and exacerbate postprandial hyperglycemia, and depending on the glucocorticoid type, dosage, and treatment; the insulin regimen may be adjusted. This varies from one patient to another and from day to day; therefore, a thorough discussion with the patient’s family and endocrinology team input is important.

## Conclusions

Optic neuritis, though an uncommon disease in pediatric patients, can present in patients with type one diabetes. Management with steroids can be challenging in these patients due to steroid-induced hyperglycemia. A multi-disciplinary team approach with neurologists, ophthalmologists, endocrinologists, and social workers is needed to ensure these patients are safely managed for their optic neuritis while minimizing steroid side effects.
